# Effect and safety of perioperative ketamine/esketamine administration on postoperative pain and depression after breast cancer surgery: a systematic review and meta-analysis

**DOI:** 10.3389/fphar.2025.1532524

**Published:** 2025-03-28

**Authors:** Xinyi Sun, Chengwei Li, Lin Xu, Xiaojie Lin, Zheng Zhang, Chunlong Lin, Jianjun Li, Penghui Wei

**Affiliations:** Department of Anesthesiology, Qilu Hospital (Qingdao), Cheeloo College of Medicine, Shandong University, Qingdao, China

**Keywords:** ketamine, esketamine, breast cancer surgery, postoperative pain, postoperative depression, meta-analysis

## Abstract

**Background:**

Patients with breast cancer experience varying degrees of pain, depression, and anxiety after surgery, which affect their postoperative recovery. Although ketamine/esketamine exhibit potential for opioid-sparing and controlling postoperative pain and depression, their effects on postoperative pain and depression remain unclear. This meta-analysis aimed to evaluate whether perioperative administration of ketamine/esketamine could reduce postoperative pain and depression, improve postoperative recovery, and reduce the incidence of adverse events in patients after breast cancer surgery.

**Material and methods:**

PubMed, Embase, Web of Science, Cochrane Library, and Clinical Trials were searched from inception until June 2, 2024 for randomized controlled trials in English language on the effect of perioperative ketamine/esketamine on postoperative pain in patients undergoing breast cancer surgery. The primary outcome was the postoperative pain score, and the secondary outcomes were the postoperative depression score, quality of postoperative recovery, incidence of adverse events, and extubation time. The standardized mean difference and 95% confidence interval (CI) were calculated for continuous outcomes, and the risk ratio and 95% CI were calculated for binary variables.

**Results:**

Seven studies involving 748 patients were included in this meta-analysis. No significant differences were found in postoperative pain scores at 2 h, 4 h, 1 day, 3 days, 7 days, and 3 months after surgery. Postoperative depression scores at 3 and 7 days after surgery were lower in the ketamine/esketamine group. The incidence of dizziness was lower in ketamine/esketamine group. No statistically significant differences were observed in postoperative depression scores at 30 days after surgery, quality of postoperative recovery at 1 and 3 days after surgery, extubation time, or the incidence of nausea, vomiting, and nightmares.

**Conclusion:**

Perioperative ketamine/esketamine administration did not significantly reduce postoperative pain in patients undergoing breast cancer surgery; however, it may reduce depression within a short period after the surgery.

**Clinical Trial Registration:**

https://www.crd.york.ac.uk/PROSPERO/display_record.php?ID=CRD42024572414, identifier CRD42024572414.

## Introduction

In 2020, breast cancer overtook lung cancer as the most common cancer in females worldwide. (Sung et al., 2021). A meta-analysis further revealed that approximately half of all women who undergo breast cancer surgery experience persistent postoperative pain, with approximately a quarter experiencing moderately to severely persistent postoperative pain. (Wang et al., 2020). Acute pain can become persistent through Sp4-dependent overexpression of transient receptor potential (TRP) channels and sustained production of inflammatory mediators (Schumacher, 2024). Studies have shown that approximately 50% of patients with acute post-operative pain will develop chronic pain. (Schumacher, 2024). Long term chronic pain and undergoing radical breast cancer surgery greatly increase the risk of postoperative depression in breast cancer patients. (Kim et al., 2017; Gohari et al., 2022). Notably, postoperative pain and depression affect patient wellbeing and are associated with a decreased quality of life, increased risk of unemployment, and increased healthcare costs. (Wang et al., 2020).

Ketamine, a racemic mixture of (S)-ketamine and (R)-ketamine, (Adams et al., 1978), has been used clinically as an anesthetic since 1970. (Dundee et al., 1970). In addition to its primary dissociative anesthetic properties, (Domino, 2010), ketamine exerts its analgesic effect by binding to the N-methyl-D-aspartate (NMDA) receptor and blocking the inward flow of calcium ions, inhibiting central sensitisation and pain signalling. (Wang K. et al., 2024; Zanos and Gould, 2018; Laskowski et al., 2011). Furthermore, ketamine may also exert antidepressant effects by affecting the Mechanistic Target of Rapamycin and Brain Derived Neurotrophic Factor (mTOR-BDNF) signalling pathway, modulating synaptic plasticity and neurotransmitter release (Zanos and Gould, 2018; Chen et al., 2024). However, the potential side effects of ketamine, including dissociative, psychotomimetic effects and cognitive impairment, limit its clinical application. (Laskowski et al., 2011; Cohen et al., 2018; Avidan et al., 2017; Shaffer et al., 2014; Zanos et al., 2018; Shinohara et al., 2021). In contrast, esketamine, the S-isomer of ketamine, exhibits a stronger affinity for NMDA receptors, requires a smaller dose for the onset of action, and has fewer side effects than ketamine. (Mion and Himmelseher, 2024). Clinical trials have demonstrated the advantages of esketamine in perioperative settings. For instance, a randomized controlled trial reported that the perioperative use of low-dose esketamine significantly reduced postoperative pain scores through anti-inflammation in elderly patients undergoing lumbar spine surgery. (Hou et al., 2025). Meanwhile, esketamine is also able to reduce the use of opioids, which is beneficial for maintaining intraoperative haemodynamic stability in patients and reducing the incidence of postoperative respiratory depression. (Hou et al., 2025). Additionally, esketamine has a faster onset of action than ketamine in the antidepressant setting. It has been shown that esketamine improves depression by inhibiting TREK-1 (TWIK-related K^+^ channel 1) channels and modulating neurotransmitters in postoperative breast cancer patients. (Xu et al., 2025). Notably, ketamine/esketamine have received considerable research attention in recent years because of their potential rapid antidepressant and analgesic effects in perioperative pain management and antidepressant applications. (Miziara et al., 2016; Su et al., 2022; Jiang et al., 2016). However, the effects of perioperative ketamine/esketamine administration on postoperative pain and depression in patients undergoing breast cancer surgery remain controversial. Moreover, the widespread perioperative use of ketamine/esketamine is limited by the uncertainty of their long-term effects and safety. (Avidan et al., 2017; Wang et al., 2019; Zhu et al., 2022). Therefore, this meta-analysis was aimed to explore the effects of ketamine and esketamine on postoperative pain and depression in patients after breast cancer surgery to guide their perioperative application.

## Material and methods

This systematic review and meta-analysis was conducted according to the recommendations of the Preferred Reporting Items for Systematic Reviews and Meta-Analyses (PRISMA) (Page et al., 2021) and Assessing the methodological quality of systematic reviews (AMSTAR) Guidelines (Shea et al., 2017) and registered in PROSPERO.

### Search strategy and eligibility criteria

PubMed, Embase, Web of Science, Cochrane Library, and Clinical Trials were systematically searched from inception until June 2, 2024 using MeSH and free-text terms. The PubMed search was performed using the following keywords: “((Esketamine [Title/Abstract]) OR (ketamine [Title/Abstract])) AND (((Breast cancer [Title/Abstract]) OR (Breast tumor [Title/Abstract])) OR (breast surgery [Title/Abstract])).” The language was restricted to English. The inclusion criteria were defined according to the PICOS framework: 1) Population: adult patients (≥18 years) undergoing breast cancer surgery; 2) Intervention: Perioperative (pre-, intra- or postoperative) single or continuous infusion of ketamine/esketamine; 3) Comparison: placebo (normal saline); 4) Outcomes: primary outcome as postoperative pain scores, secondary outcomes including depression scores, quality of recovery, adverse events, and extubation time; 5) Study design: only randomized controlled trials (RCTs).

### Exclusion criteria

Non-RCTs, case reports, conference abstracts, comments, systematic reviews, and studies involving animal experiments, non-intubation general anesthesia, pediatric surgery, ketamine/esketamine as an adjuvant to regional anesthesia, and a combination of ketamine/esketamine and bupivacaine, lidocaine, or dexmedetomidine, as well as studies that did not report postoperative pain scores, were excluded.

### Study selection and data collection

Two authors independently selected eligible studies and extracted data based on the predefined study selection criteria and clinical endpoints. Disagreements between the two authors were resolved through discussion with another senior researcher. The data, including first author/year, ASA grade, sample size, age, ketamine/esketamine administration details (dosage and timing), and country of origin, were extracted from the selected studies. The primary outcome of the study was the postoperative visual analog scale score for pain, whereas the secondary outcomes were the postoperative depression scores, quality of postoperative recovery, risk of adverse events (such as nausea, vomiting, dizziness, and nightmares), and extubation time.

### Assessment of risk of bias

The revised Cochrane Risk of Bias 2 (RoB 2) tool was used to assess the quality of the included RCTs in five domains: randomization process, deviations from intended interventions, missing outcome data, measurement of outcomes, and selective reporting by two authors independently, and the risk of overall bias was graded as high, unclear, or low (Higgins et al., 2011; Nejadghaderi et al., 2024). Disagreements were resolved through discussion with a third author.

### Statistical analysis

Statistical analyses were performed using Review Manager 5.3 (Cochrane Collaboration, Oxford, UK) and STATA 16.0. The chi-square and I^2^ tests were employed for all meta-analyses to evaluate statistical heterogeneity, which was classified as low (I^2^ < 50%), moderate (I^2^ = 50–75%), and high (I^2^ > 75%). (Melsen et al., 2014). The choice between fixed-effect and random-effects models was based on both statistical and clinical heterogeneity (Melsen et al., 2014; Borenstein et al., 2010). A random-effects model was applied if significant heterogeneity was detected (I^2^ > 50% or p < 0.05), accounting for variability across studies in surgical techniques, dosing regimens, and outcome assessment (Melsen et al., 2014; Borenstein et al., 2010). Otherwise, a fixed effects model was used. This approach aligns with recommendations for meta-analyses with heterogeneous populations or interventions. Standardized mean difference (SMD) and 95% confidence interval (CI) were calculated for continuous outcomes, whereas risk ratio (RR) with 95% CI were used to compare binary variables. The median and interquartile range (IQR) or the median and 95% CI of continuous data were converted to mean and standard deviation (SD) based on the method described by Wan et al. (Wan et al., 2014) Statistical significance was set at p < 0.05. A sensitivity analysis was performed to evaluate the stability of the primary outcomes.

### Assessment of publication bias and quality of evidence

If the number of included studies is greater than 10, we planned to use funnel plots to assess the potential for publication bias. (Sterne et al., 2011). We used the Grading of Recommendations, Assessment, Development, and Evaluations (GRADE) framework to assess the quality and strength of the evidence base. (Guyatt et al., 2008; Granholm et al., 2019). All assessments were performed independently by two investigators, followed by discussions to reach a consensus.

## Results

### Search results

**FIGURE 1:**
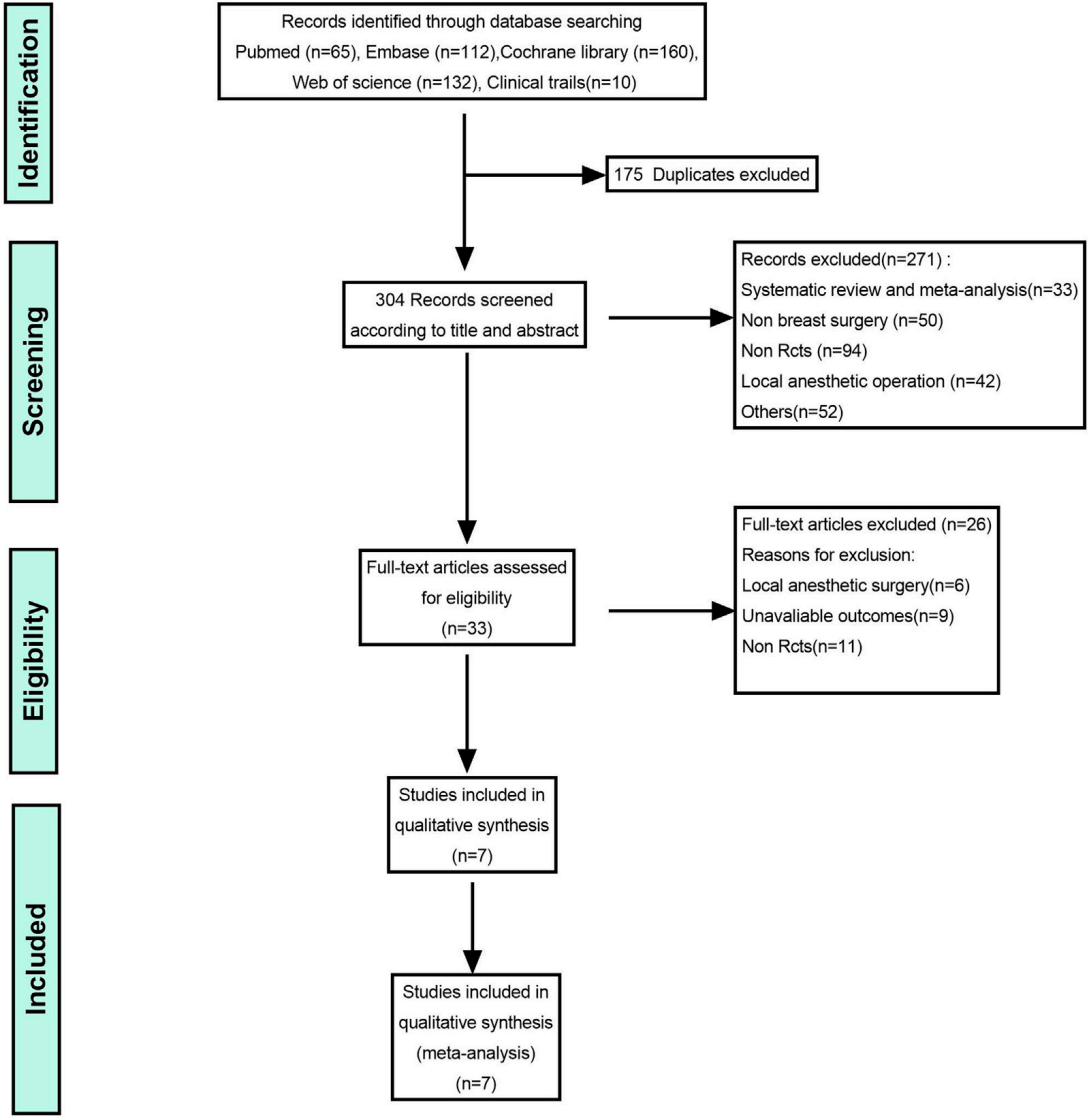
Flowchart of the search strategy used to identify eligible randomized controlled trials.

Initially, 479 potentially eligible studies were identified. After removing 175 duplicate records, 304 studies were screened based on their titles and abstracts, and 33 full-text articles were evaluated for their eligibility. After excluding 11 non-RCT studies, 6 studies that included local anesthetic nerve blocks, and 9 studies without primary endpoints, 7 studies were finally included. (Zhu et al., 2022; Mahran and Hassan, 2015; Ranran et al., 2017; Kang et al., 2020; Liu et al., 2021; Zhao et al., 2021; Wang H. et al., 2024). A flowchart of our study selection process is presented in Figure 1.

### Study characteristics

Overall, the included studies involved 748 patients, of which 390 and 358 received ketamine/esketamine and NS as a control, respectively. The characteristics of the included studies are summarized in Supplementary Table 1. Of the seven RCT studies included, five (Zhu et al., 2022; Ranran et al., 2017; Kang et al., 2020; Zhao et al., 2021; Wang H. et al., 2024) were modified radical mastectomies and the other two (Mahran and Hassan, 2015; Liu et al., 2021) did not describe the specific surgical procedure for breast cancer. Most studies enrolled patients with an ASA classification of I–II, (Zhu et al., 2022; Mahran and Hassan, 2015; Ranran et al., 2017; Kang et al., 2020; Zhao et al., 2021; Wang H. et al., 2024), and only one study included patients with an ASA physical status of III. (Liu et al., 2021). Three (Zhu et al., 2022; Liu et al., 2021; Wang H. et al., 2024) and four (Mahran and Hassan, 2015; Ranran et al., 2017; Kang et al., 2020; Zhao et al., 2021) studies used esketamine and ketamine, respectively. Five studies (Zhu et al., 2022; Ranran et al., 2017; Liu et al., 2021; Zhao et al., 2021; Wang H. et al., 2024) involved intraoperative administration of ketamine/esketamine, and two studies (Mahran and Hassan, 2015; Kang et al., 2020) involved preoperative and intraoperative administration. In addition, the dosing regimen differed in each study, with loading doses ranging from 0.125 to 0.5 mg/kg and infusion rates from 0.002 to 0.25 mg/kg/h. Four studies used the postoperative VAS score, (Mahran and Hassan, 2015; Ranran et al., 2017; Liu et al., 2021; Wang H. et al., 2024), and three studies used the postoperative numeric rating scale (NRS) pain score. (Zhu et al., 2022; Kang et al., 2020; Zhao et al., 2021). Only three studies involved postoperative depression scoring using the Hamilton Depression Scale (Ranran et al., 2017; Liu et al., 2021) and the Hospital Anxiety and Depression Scale. (Zhao et al., 2021). Three studies assessed the quality of postoperative recovery using three different scores: 40-Item Quality of Recovery scale, (Zhao et al., 2021), quality of recovery-15 scores, (Zhu et al., 2022), and the Patient Health Questionnaire-9 scores. (Wang H. et al., 2024).

### Risk of bias in included studies

**FIGURE 2:**
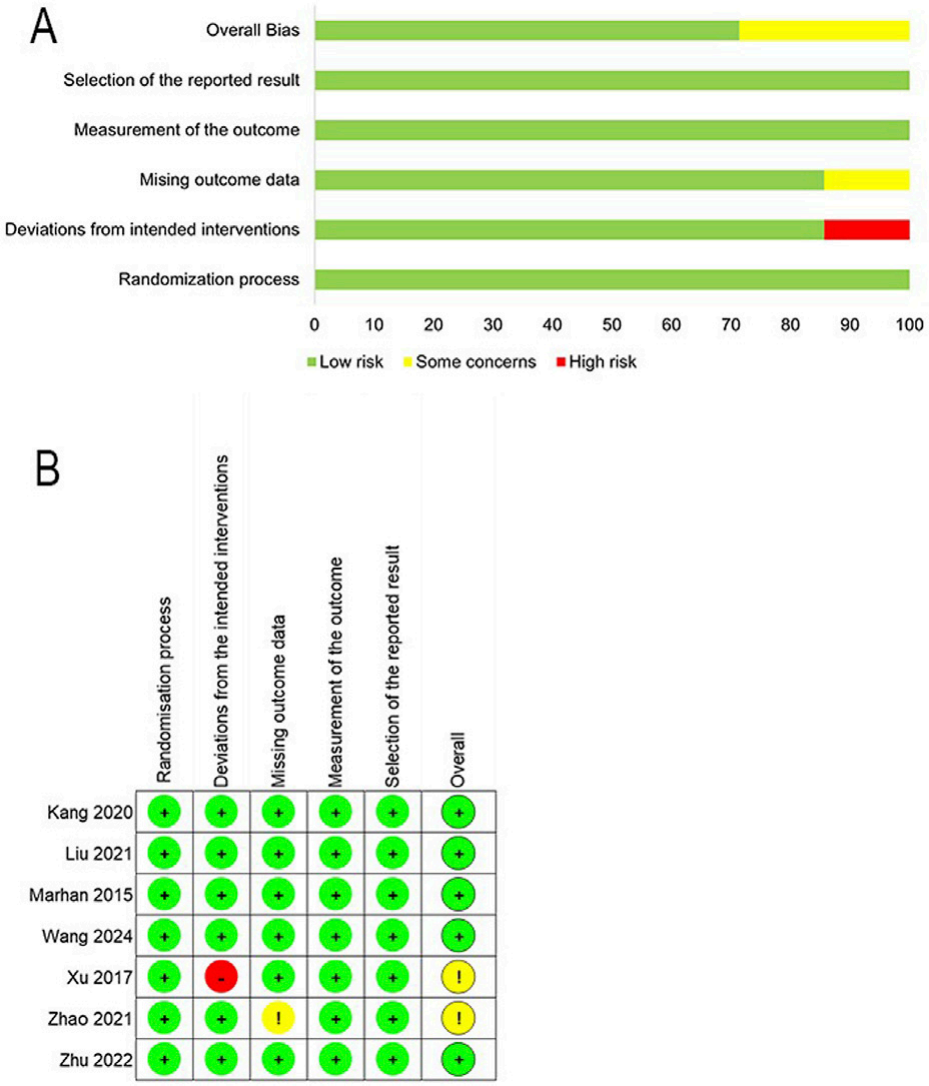
Risk of bias assessment. **(A)** Risk-of-bias summary. **(B)** Risk of bias in individual studies. Risk of bias methods: (+), a low risk of bias; (?), unclear risk of bias; and (−), high risk of bias.

Figure 2 displays the quality assessment results of the included studies, conducted according to the revised Cochrane RoB 2 tool. In total, there are 5 studies with a low overall risk of bias, which indicates reliable methodologies and findings (Wang et al., 2020; Zhu et al., 2022; Mahran and Hassan, 2015; Kang et al., 2020; Liu et al., 2021). One study raised some concerns due to missing outcome data (Zhao et al., 2021), while one study was rated as unclear one study’s risk of bias was rated unclear for deviations from the intended interventions (Ranran et al., 2017).

### Pooled results of included studies

#### Primary outcome

**FIGURE 3:**
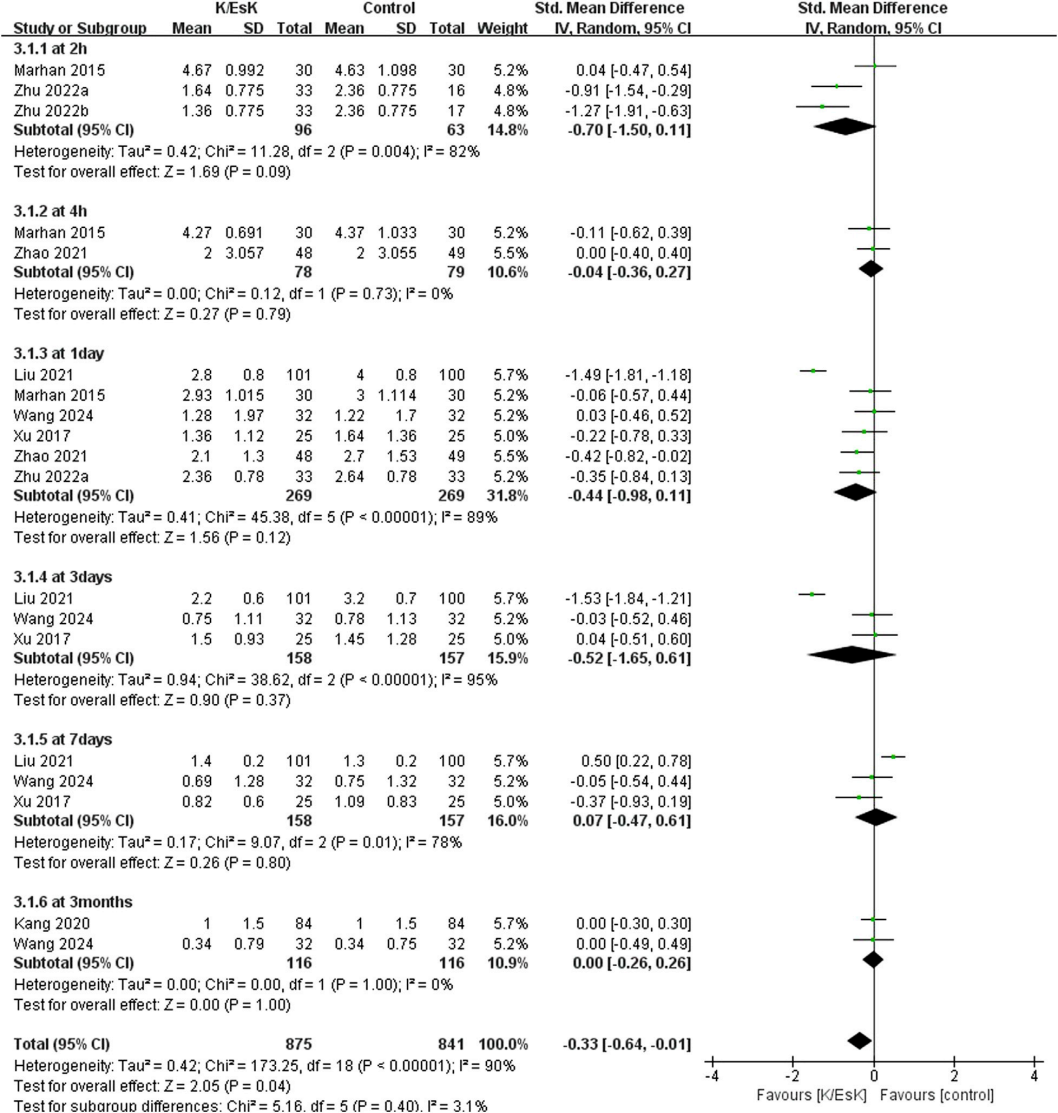
Forest plot of the effect of perioperative administration of ketamine/esketamine (k/esk) on postoperative pain scores within 3 months of surgery. CI, confidence interval; df, degrees of freedom; Std, standardized.

**FIGURE 4:**
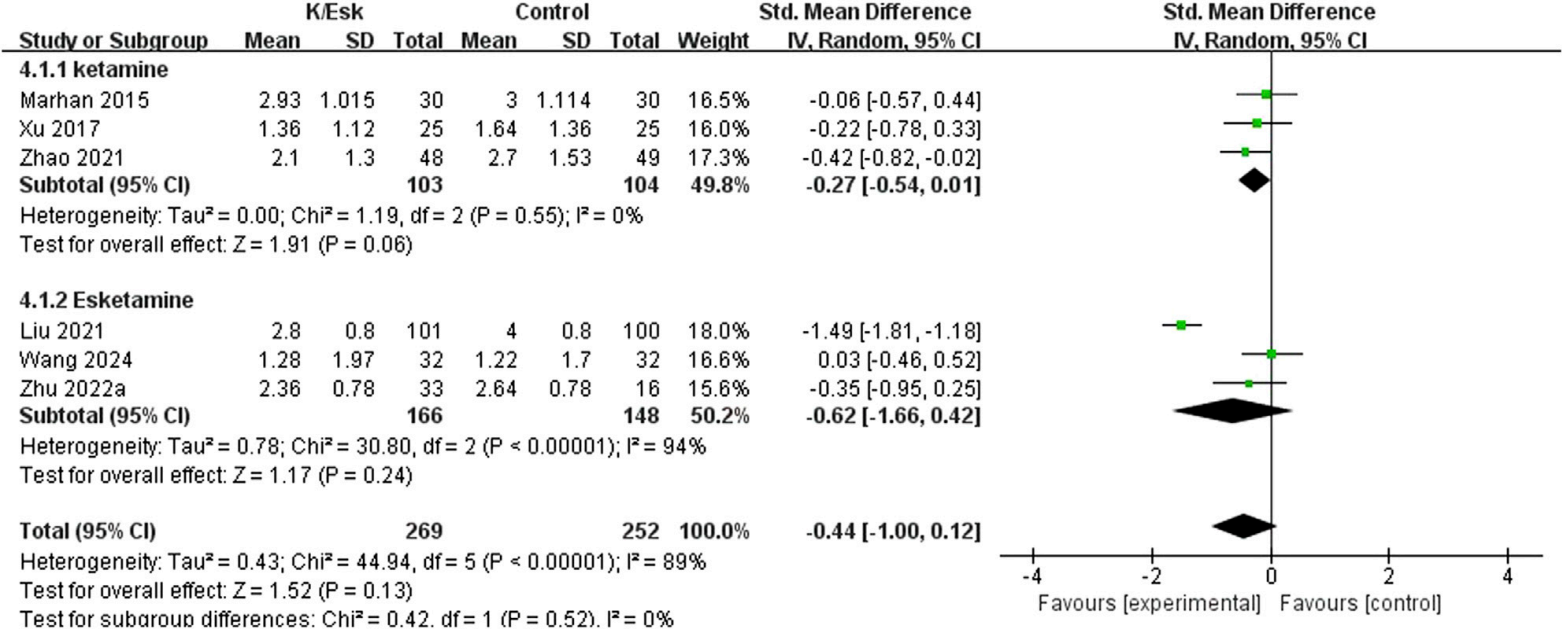
Forest plot of the subgroup analysis of the effect of perioperative administration of ketamine/esketamine (k/esk) on postoperative pain scores at 1 day after surgery. CI, confidence interval; df, degrees of freedom; Std, standardized.

Seven studies reported postoperative pain scores, (Zhu et al., 2022; Mahran and Hassan, 2015; Ranran et al., 2017; Kang et al., 2020; Liu et al., 2021; Zhao et al., 2021; Wang H. et al., 2024), and three of them reported NRS scores as medians (IQRs), (Zhu et al., 2022; Kang et al., 2020; Zhao et al., 2021), which were converted into means ± SDs. The results revealed that ketamine/esketamine did not reduce pain scores in patients with breast cancer at 2 h (SMD: −0.70, 95% CI: −1.50 to 0.11, p = 0.09, I^2^ = 82%), 4 h (SMD: −0.04, 95% CI: −0.36 to 0.27, p = 0.79, I^2^ = 0%), 1 day (SMD: −0.44, 95% CI: −0.98 to 0.11, p = 0.12, I^2^ = 89%), 3 days (SMD: −0.52, 95% CI: −1.65 to 0.61, p = 0.37, I^2^ = 95%), 7 days (SMD: 0.07, 95% CI: −0.47 to 0.61, p = 0.80, I^2^ = 78%) and 3 months (SMD: 0.00, 95% CI: −0.26 to 0.26, p = 1.00, I^2^ = 0%) after surgery (Figure 3). We also analyzed the postoperative pain scores according to the drug type and found no statistically significant difference between ketamine and esketamine for reducing postoperative pain scores at 1 day after surgery (SMD: −0.44, 95% CI: −1.00 to 0.12, p = 0.52, I^2^ = 0%) (Figure 4).

#### Secondary outcomes

**FIGURE 5:**
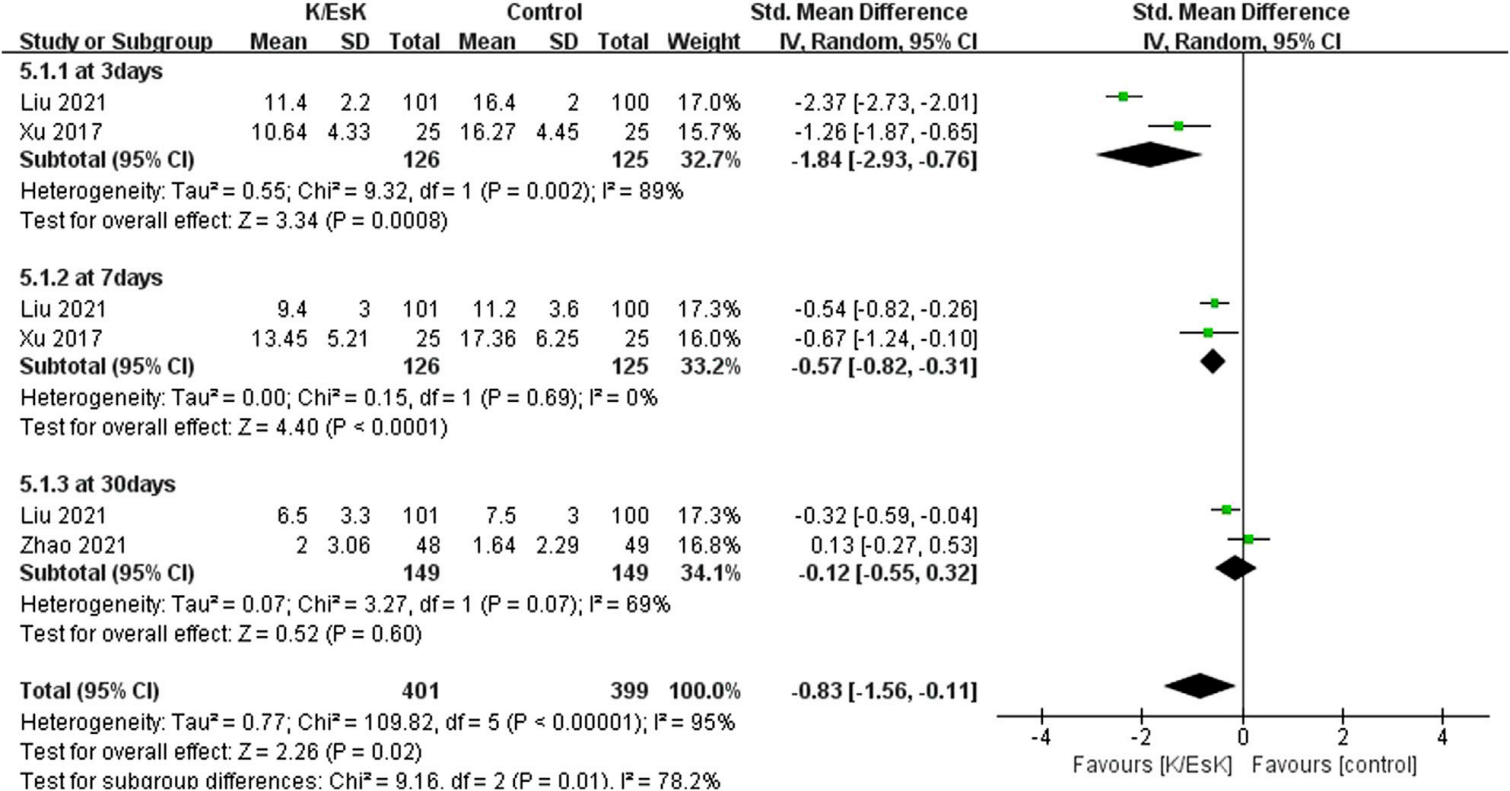
Forest plot of the effect of perioperative administration of ketamine/esketamine (k/esk) on postoperative depression scores within 30 days of surgery. CI, confidence interval; df, degrees of freedom; Std, standardized.

**FIGURE 6:**
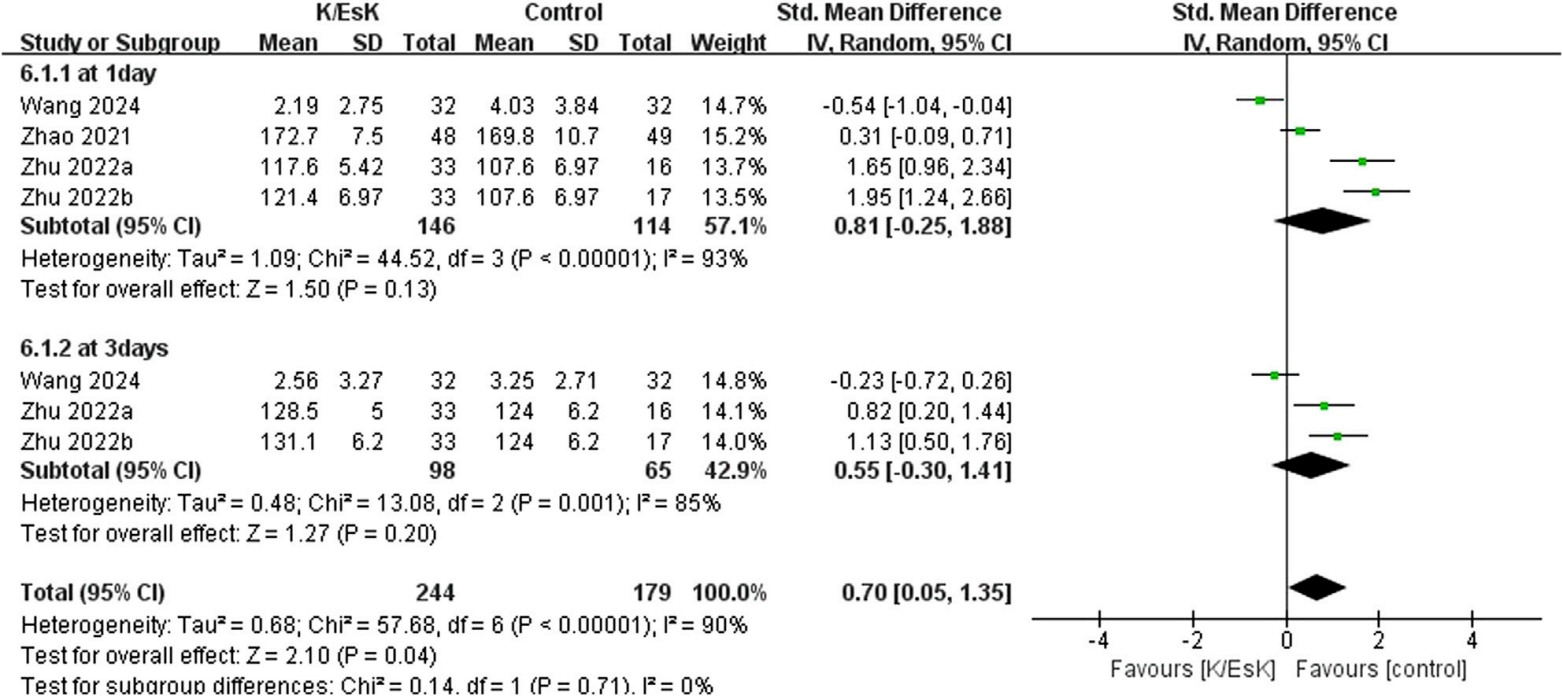
Forest plot of the effect of perioperative administration of ketamine/esketamine (k/esk) application on the quality of postoperative recovery within 3 days after surgery. CI, confidence interval; df, degrees of freedom; Std, standardized.

**FIGURE 7:**
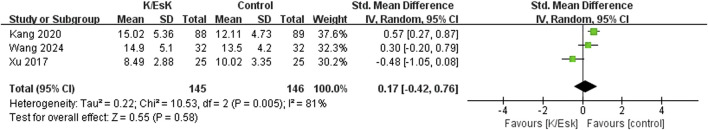
Forest plot of the effect of perioperative administration of ketamine/esketamine (k/esk) on extubation time after surgery. CI, confidence interval; df, degrees of freedom; Std, standardized.

**FIGURE 8:**
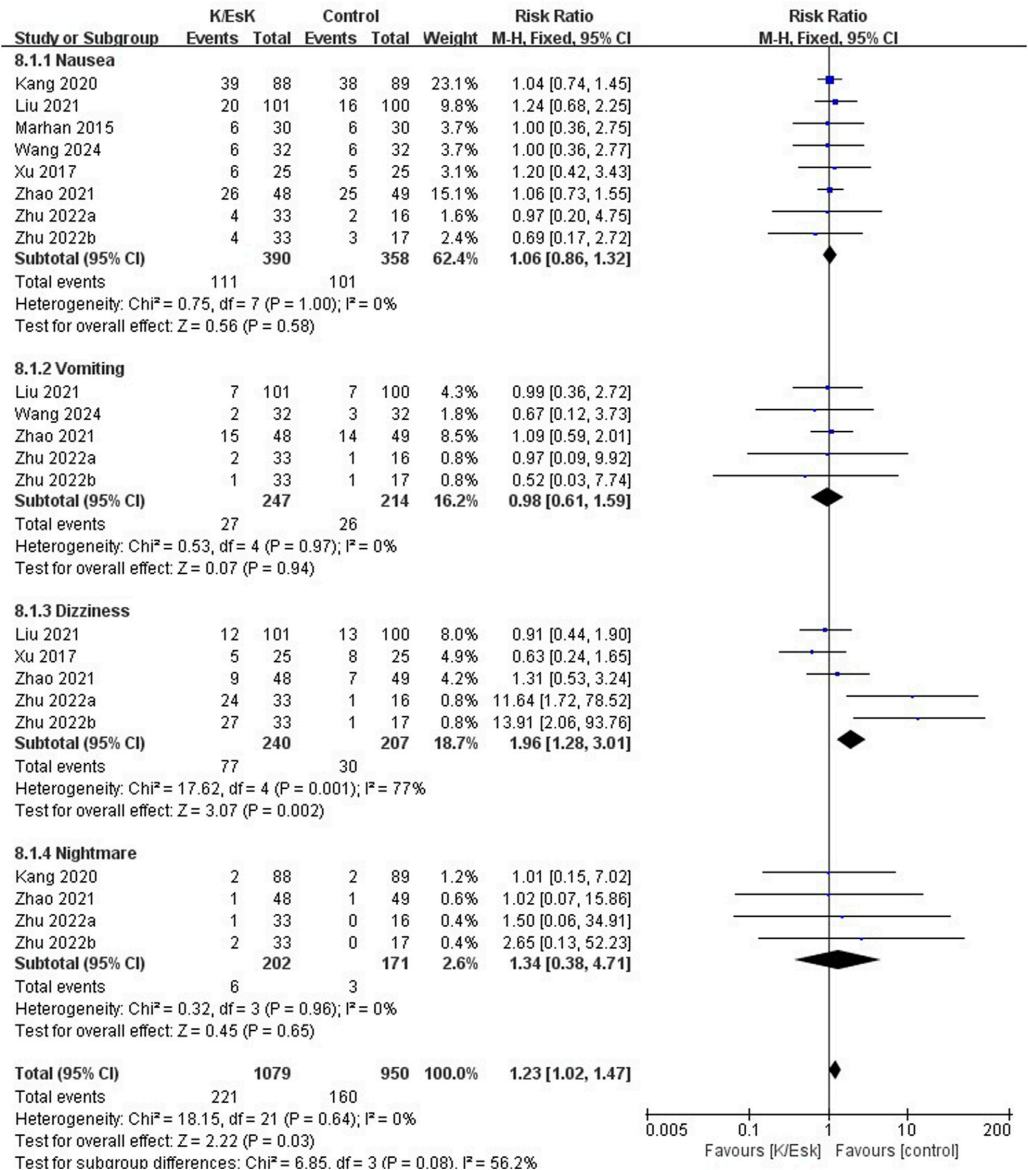
Forest plot of the incidence of postoperative adverse effects following perioperative ketamine/esketamine (k/esk) administration. CI, confidence interval; df, degrees of freedom.

Three studies reported postoperative depression scores (Ranran et al., 2017; Liu et al., 2021; Zhao et al., 2021), with a total sample size of 348 patients (174 in the ketamine/eketamine group and 174 in the control group), one of which was reported at as medians (IQRs), and these data were converted into means ± SDs. (Zhao et al., 2021). We also performed an analysis based on different postoperative times for the postoperative depression scores. Notably, the postoperative depression scores of patients in the ketamine/esketamine group were lower than those of the control group at 3 days (SMD: −1.84, 95% CI: −2.93 to −0.76, p < 0.001, I^2^ = 89%) and 7 days (SMD: −0.57, 95% CI: −0.82 to −0.31, p < 0.001, I^2^ = 0%) after surgery. However, no statistically significant difference was observed in postoperative depression scores between the two groups at 30 days after surgery (SMD: −0.12, 95% CI: −0.55 to 0.32, p = 0.60, I^2^ = 69%) (Figure 5). One study presented the results for the quality of postoperative recovery as medians (IQRs), which were converted into means ± SDs. (Zhu et al., 2022). No statistically significant difference was observed in the quality of recovery at 1 day (SMD: 0.81, 95% CI: −0.25 to 1.88, p = 0.13, I^2^ = 93%) and 3 days (SMD: 0.55, 95% CI: −0.30 to 1.41, p = 0.20, I^2^ = 85%) after surgery (Figure 6). Three studies reported data for extubation time, (Ranran et al., 2017; Kang et al., 2020; Wang H. et al., 2024), and the results revealed no significant difference (SMD: 0.17, 95% CI: −0.42 to 0.76, p = 0.58, I^2^ = 81%) between the groups. (Figure 7). The pooled results revealed a lower incidence of dizziness in ketamine/esketamine group compared to the NS group (RR: 1.96, 95% CI: 1.28 to 3.01, p = 0.002, I^2^ = 77%) (Figure 8). No statistical differences were found between the groups regarding the incidence of nausea (RR: 1.06, 95% CI: 0.86 to 1.32, p = 0.58, I^2^ = 0%), vomiting (RR: 0.98, 95% CI: 0.61, 1.59, p = 0.94, I^2^ = 0%), and nightmares (RR: 1.34, 95% CI: 0.38 to 4.71, p = 0.65, I^2^ = 0%) (Figure 8).

### Sensitivity analysis

**FIGURE 9:**
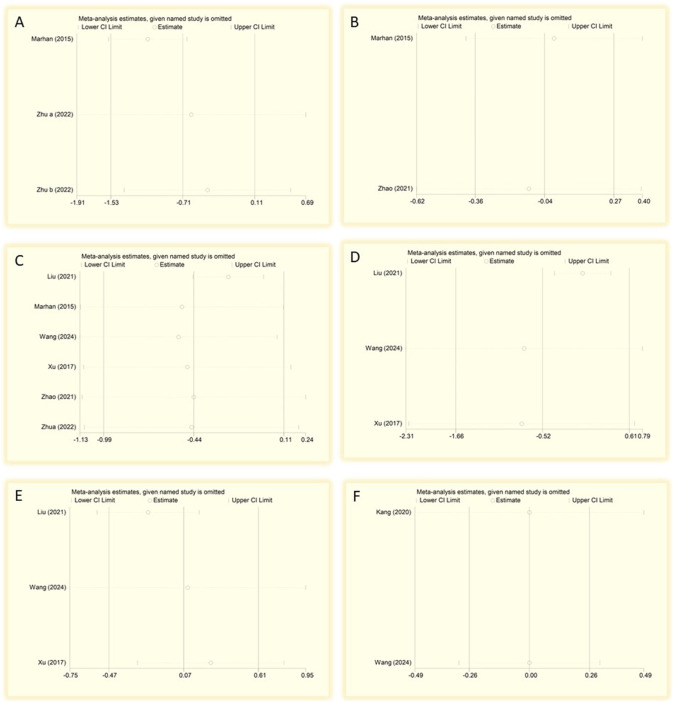
Sensitivity analysis of postoperative pain scores at different time points after surgery. **(A)** 2 h, **(B)** 4 h, **(C)** 3 months, **(D)** 1 day, **(E)** 3 days, and **(F)** 7 days after surgery. CI, confidence interval; df, degrees of freedom; Std, standardized.

Sensitivity analyses performed to evaluate the stability of the primary outcomes. The results revealed that one study had a significant impact on the stability of ketamine/esketamine on pain scores at 2 h after surgery (Mahran and Hassan, 2015) (Figure 9A). Furthermore, the results of 4 h and 3 months postoperative pain scores were more stable (Figures 9B,C). Additionally, a greater effect of ketamine/esketamine on postoperative pain scores at 1 day, 3 days, and 7 days after surgery was observed in the study of Liu (Liu et al., 2021) (Figures 9D–F).

### Publication bias test

We did not perform a publication bias test, as we included <10 studies.

### Quality of the evidence

According to the GRADE, for the primary outcome, the quality of evidence for pain scores at 4 h and 3 months after surgery was considered “moderate,” the quality of evidence for pain scores at 2 h, and 1 day after surgery was considered “low,” and the quality of evidence for pain scores at other times was considered “very low.” In addition, for secondary outcomes, the quality of evidence for vomiting was considered “high”, and the quality of evidence for nausea and nightmare was considered “moderate”. The evidence of depression scores at 3 days after surgery and the extubation time was considered “very low”. The quality of evidence for the remaining secondary outcomes was considered “low”. (Supplementary Table 1).

## Discussion

This meta-analysis revealed that perioperative administration of ketamine/esketamine prevents depressive symptoms in the early postoperative period to a certain extent; however, their effectiveness in reducing postoperative pain, promoting the quality of recovery, and reducing adverse effects was limited. This phenomenon may be related to the complexity of the mechanism of action of these drugs, individual patient differences, and their effects on the body. Our findings align with previous studies demonstrating the antidepressant effects of ketamine/esketamine in perioperative settings. However, unlike prior research, which primarily focused on analgesic efficacy, our study highlights the potential of these drugs in preventing early-onset depressive symptoms. This distinction is clinically significant, as postoperative depression is often underdiagnosed and undertreated. While ketamine/esketamine is widely used, our findings provide additional evidence supporting their role in managing postoperative mental health, particularly in high risk populations.

Breast cancer is one of the most common malignant tumors among females, which affects the physical and mental health of patients. (Sung et al., 2021). Although modified radical mastectomy is considered the most effective treatment for breast cancer, most patients experience different degrees of postoperative pain as well as emotional disturbances such as anxiety, depression, and fear because of surgical resection, nerve damage, and inflammatory stimulation. This in turn reduces patient satisfaction and leads to poor wound healing, thus affecting postoperative recovery and the quality of life of the patients. (Zhu et al., 2022; Wang H. et al., 2024; Werner and Kongsgaard, 2014).

Ketamine, as an NMDA receptor antagonist, has been used in clinical anesthesia for many years because of its powerful sedative and analgesic effects. Esketamine, the S-(+) enantiomer of ketamine with all substituents on the same side and a stereochemically chiral center, exhibits approximately three to four times greater affinity for the NMDA receptors than that of R-ketamine, thus resulting in a higher bioactivity and fewer adverse effects, particularly as an analgesic and antidepressant. (Wang et al., 2019; Li et al., 2022). Ketamine and esketamine act as noncompetitive antagonists of NMDA receptors, and their pharmacological properties mainly involve the modulation of the central nervous system. Their mechanism of action may also be related to neuroplasticity and altered mood states in addition to modulating pain perception. (Wang et al., 2019; Zhu et al., 2022; Autry et al., 2011; Li et al., 2010).

Sustained injurious stimuli can lead to pain sensitization by activating NMDA receptors. The mechanism of ketamine-induced antinociceptive sensitization primarily involves noncompetitive antagonism of NMDA receptors. Previous studies have reported the perioperative use of esketamine in relieving postoperative pain and reducing opioid consumption. (Miziara et al., 2016; Su et al., 2022). A meta-analysis reported that the perioperative use of ketamine/eketamine was associated with improvements in early subjective quality of recovery, pain severity, and psychological symptoms without increasing the likelihood of adverse events. (Hung et al., 2024). However, Brinck et al. found that the intraoperative administration of esketamine did not reduce postoperative pain or oxycodone consumption during lumbar fusion surgery, which is consistent with the findings of our meta-analysis. (Brinck et al., 2021). This may be attributed to several factors, including, but not limited to, type of surgery, drug dose, route of administration, age, and individual differences in pain thresholds. (Laskowski et al., 2011; Avidan et al., 2017; Zhao et al., 2021). Notably, intraoperative ketamine application improved postoperative depression scores and elevated serum BDNF levels in patients undergoing elective orthopaedic surgery. (Jiang et al., 2016). Ketamine/esketamine can rapidly increase presynaptic glutamate release and BDNF synthesis by antagonizing NMDA receptors, (Autry et al., 2011; Li et al., 2010), which in turn promotes structural synaptic connectivity, resulting in a prolonged antidepressant effect. (Liu et al., 2012; Li et al., 2011). Tu et al. reported that eketamine administration during the induction of anesthesia reduced the perioperative inflammatory response and promoted the recovery of postoperative cognitive function in older patients after surgery. (Tu et al., 2021). However, the analgesic and antidepressant effects of ketamine and esketamine are not exclusively dependent on NMDA receptor antagonists and may involve multiple metabolites and mechanisms. (Zanos and Gould, 2018; Zanos et al., 2016).

Although ketamine and esketamine may potentially improve postoperative pain and early depression, the conclusion remain inconsistent, and their potential adverse effects and long-term safety issues limit their widespread perioperative use. (Shaffer et al., 2014). Perioperative esketamine administration significantly reduced pain intensity at 24 h postoperatively but increased Bispectral Index values and the incidence of drowsiness. (Zhu et al., 2022). In addition, a multicenter study found that the perioperative ketamine administration did not improve postoperative delirium in older adults after major surgery and increased the incidence of postoperative hallucinations and nightmares, thus inducing negative experiences. (Avidan et al., 2017). Therefore, clinicians should thoroughly assess the risks and benefits of ketamine and esketamine in perioperative management and develop individualized perioperative regimens to ensure patient safety.

This meta-analysis provides evidence supporting the potential of ketamine and esketamine to improve early postoperative depression in patients with breast cancer. Nevertheless, this study also has some limitations. First, only seven studies with relatively small sample sizes were included in our meta-analysis, which may have affected the statistical validity. Future larger trials and longer follow-up times are needed to further validate the findings of this meta-analysis. Second, the baseline characteristics of most studies were well-balanced, (Zhu et al., 2022; Mahran and Hassan, 2015; Ranran et al., 2017; Kang et al., 2020; Liu et al., 2021; Wang H. et al., 2024), and one study exhibited comparable baseline characteristics, which might have affected the accuracy of our results. (Zhao et al., 2021). Third, the measurement method of postoperative pain scores differed among the studies, with four (Mahran and Hassan, 2015; Ranran et al., 2017; Liu et al., 2021; Wang H. et al., 2024) and three (Zhu et al., 2022; Kang et al., 2020; Zhao et al., 2021) studies using the VAS and NRS scores, respectively, which may have affected the accuracy of our results. Fourth, four of the seven studies did not include postoperative depression scores as the primary outcome (Zhu et al., 2022; Mahran and Hassan, 2015; Kang et al., 2020; Wang H. et al., 2024); therefore, the data we extracted might be the occasional findings of these studies. Fifth, there may be heterogeneity in the type of surgery included in the study (modified radical versus breast-conserving surgery), and the severity of postoperative pain and depression may vary depending on the invasiveness of the surgery. Future studies need to be further stratified to analyse the effect of type of surgery on outcomes. Finally, we could not explore the mechanisms for improving postoperative depression and pain, as only one study assessed the perioperative serum BDNF and 5-hydroxytryptamine levels. (Liu et al., 2021).

Multicenter studies with larger sample sizes should be conducted in the future to improve the reliability and general applicability of the results regarding the use of ketamine/esketamine in postoperative management. In addition, exploring more precise strategies for the use of ketamine/esketamine in patients undergoing breast cancer surgery, such as optimal dosage, timing of administration, and patient screening criteria, would help further optimize their clinical use. Moreover, long-term follow-up studies may help assess the long-term effects of these drugs on postoperative pain and depression, as well as their combined effects on the quality of life of the patients.

## Conclusion

Perioperative ketamine/esketamine administration did not significantly reduce postoperative pain in patients after breast cancer surgery; however, ketamine/esketamine may reduce depression in patients within a short period after the surgery.

## Data Availability

The original contributions presented in the study are included in the article/Supplementary Material, further inquiries can be directed to the corresponding authors.
