# Supplementary material for: Effect and safety of perioperative ketamine/esketamine administration on postoperative pain and depression after breast cancer surgery: a systematic review and meta-analysis
Source: Front Pharmacol. 2025 Mar 28;16:1532524. doi: 10.3389/fphar.2025.1532524 (PMC11985805; doi:10.3389/fphar.2025.1532524)
Supplement: Supplementary file 1 [file Table1.docx]

**GRADE**

| **Quality assessment** | | | | | | | **No of patients** | | **Effect** | | **Quality** | **Importance** |
| --- | --- | --- | --- | --- | --- | --- | --- | --- | --- | --- | --- | --- |
|  |  |  |  |  |  |  |  |  |  |  |  |  |
| **No of studies** | **Design** | **Risk of bias** | **Inconsistency** | **Indirectness** | **Imprecision** | **Other considerations** |  |  | **Relative**  **(95% CI)** | **Absolute** |  |  |
| **Pain scores at 2 h after surgery** | | | | | | | | | | | | |
| 3 | randomised trials | no serious risk of bias | serious^1^ | no serious indirectness | serious^2^ | none | 96 | 63 | - | SMD: -0.70, 95% CI: -1.50 to 0.11 | ⊕⊕OO  LOW | CRITICAL |
| **Pain scores at 4 h after surgery** | | | | | | | | | | | | |
| 2 | randomised trials | serious^3^ | no serious inconsistency | no serious indirectness | serious^2^ | none | 78 | 79 | - | SMD: -0.04, 95% CI: -0.36 to 0.27 | ⊕⊕OO  LOW | CRITICAL |
| **Pain scores at 1 day after surgery** | | | | | | | | | | | | |
| 6 | randomised trials | serious^4^ | serious^1^ | no serious indirectness | no serious imprecision^2^ | none | 269 | 269 | - | SMD: -0.44, 95% CI: -0.98 to 0.11 | ⊕⊕OO  LOW | CRITICAL |
| **Pain scores at 3 days after surgery** | | | | | | | | | | | | |
| 3 | randomised trials | serious^3^ | serious^1^ | no serious indirectness | serious^2^ | none | 158 | 157 | - | SMD: -0.52, 95% CI: -1.65 to 0.61 | ⊕OOO  VERY LOW | CRITICAL |
| **Pain scores at 7 days after surgery** | | | | | | | | | | | | |
| 3 | randomised trials | serious^3^ | serious^1^ | no serious indirectness | serious^2^ | none | 158 | 157 | - | SMD: 0.07, 95% CI: -0.47 to 0.61 | ⊕OOO  VERY LOW | CRITICAL |
| **Pain scores at 3 months after surgery** | | | | | | | | | | | | |
| 2 | randomised trials | no serious risk of bias | no serious inconsistency | no serious indirectness | serious^2^ | none | 116 | 116 | - | SMD: 0.00, 95% CI: -0.26 to 0.26 | ⊕⊕⊕O  MODERATE | CRITICAL |
| **Depression scores at 3 days after surgery** | | | | | | | | | | | | |
| 2 | randomised trials | serious^3^ | serious^1^ | no serious indirectness | serious^2^ | none | 126 | 125 | - | SMD: -1.84, 95% CI: -2.93 to -0.76 | ⊕OOO  VERY LOW | CRITICAL |
| **Depression scores at 7 days after surgery** | | | | | | | | | | | | |
| 2 | randomised trials | serious^3^ | no serious inconsistency | no serious indirectness | serious^2^ | none | 126 | 125 | - | SMD: -0.57, 95% CI: -0.82 to -0.31 | ⊕⊕OO  LOW | CRITICAL |
| **Depression scores at 30 days after surgery** | | | | | | | | | | | | |
| 2 | randomised trials | serious^3^ | serious^1^ | no serious indirectness | serious^2^ | none | 149 | 149 | - | SMD: -0.12, 95% CI: -0.55 to 0.32 | ⊕OOO  VERY LOW | CRITICAL |
| **Quality of recovery at 1 day after surgery** | | | | | | | | | | | | |
| 4 | randomised trials | serious^3^ | serious^1^ | no serious indirectness | serious^2^ | none | 146 | 114 | - | SMD: 0.81, 95% CI: -0.25 to 1.88 | ⊕OOO  VERY LOW | IMPORTANT |
| **Quality of recovery at 3 days after surgery** | | | | | | | | | | | | |
| 3 | randomised trials | no serious risk of bias | serious^1^ | no serious indirectness | serious^2^ | none | 98 | 65 | - | SMD: 0.55, 95% CI: -0.30 to 1.41 | ⊕⊕OO  LOW | IMPORTANT |
| **Nausea** | | | | | | | | | | | | |
| 8 | randomised trials | serious^4^ | no serious inconsistency | no serious indirectness | no serious imprecision | none | 111/390  (28.5%) | 101/358  (28.2%) | RR 1.06 (0.86 to 1.32) | 17 more per 1000 (from 39 fewer to 90 more) | ⊕⊕⊕O  MODERATE | IMPORTANT |
| **Vomiting** | | | | | | | | | | | | |
| 5 | randomised trials | serious^3^ | no serious inconsistency | no serious indirectness | no serious imprecision | none | 27/247  (10.9%) | 26/214  (12.1%) | RR 0.98 (0.61 to 1.59) | 2 fewer per 1000 (from 47 fewer to 72 more) | ⊕⊕⊕O  MODERATE | IMPORTANT |
| **Dizziness** | | | | | | | | | | | | |
| 5 | randomised trials | serious^4^ | serious^1^ | no serious indirectness | no serious imprecision | none | 77/240  (32.1%) | 30/207  (14.5%) | RR 1.96 (1.28 to 3.01) | 139 more per 1000 (from 41 more to 291 more) | ⊕⊕OO  LOW | IMPORTANT |
| **Nightmare** | | | | | | | | | | | | |
| 4 | randomised trials | serious^3^ | no serious inconsistency | no serious indirectness | serious^2^ | none | 6/202  (3%) | 3/171  (1.8%) | RR 1.34 (0.38 to 4.71) | 6 more per 1000 (from 11 fewer to 65 more) | ⊕⊕OO  LOW | IMPORTANT |

^1^ The heterogeneity is high.

^2^ The sample size is small.

^3^ One study is considered to have a high risk of bias.

^4^ Two studies are considered to have a high risk of bias.
